# A novel submucosa nodule of the rectum: A case of the ectopic prostatic tissue outside the urinary tract

**DOI:** 10.12669/pjms.296.4196

**Published:** 2013

**Authors:** Sheng Dai, Xuefeng Huang, Weifang Mao

**Affiliations:** 1 Sheng Dai, MD, Department of the Colorectal Surgery, Sir Run Run Shaw Hospital, School of Medicine, Zhejiang University, P.R. China.; 2 Xuefeng Huang, PhD, MD, Department of the Colorectal Surgery, Sir Run Run Shaw Hospital, School of Medicine, Zhejiang University, P.R. China.; 3 Weifang Mao, MD, Department of the Colorectal Surgery, Sir Run Run Shaw Hospital, School of Medicine, Zhejiang University, P.R. China.

**Keywords:** Ectopic prostatic tissue, Rectal submucosa, Immunohistochemical staining, Prostate specific antigen, Embryogenesis

## Abstract

Ectopic prostatic tissue is an underreported entity, which is found most commonly in the lower male genitourinary tract, and ectopic prostate tissue outside the urinary tract is even rarer. Our patient was a  unique case of ectopic prostatic tissue within submucosa of the rectum. The patient presented with rectal bleeding, and a firm, round solid submucosa nodule found in the anterior rectum at digital rectal examination, it was 1cm in diameter and 5cm above the anal verge. The size and submucosa location of this nodule were confirmed by the colonoscopy and MRI. After being removed surgically, the histopathology of the specimen sections possessed typical prostatic acini and stroma, meanwhile the immunohistochemical staining for prostate specific antigen confirmed its’ prostatic nature. It is the first case to date, which involves the mural of rectum. We hypothesizes that the etiologies of ectopic prostatic tissue within the submucosa rectum attribute to embryogenetic abnormality.

## INTRODUCTION

Ectopic prostatic tissue is a relatively uncommon but interesting phenomenon which is most commonly encountered in the lower male urinary tract.^1^^,^^2^ However ectopic prostate tissue outside the urinary tract is even a rarer occurrence. Ectopic prostatic tissue is usually found incidentally but has also been a cause of lower gastrointestinal bleeding, obstructive symptoms, or a retroperitoneal mass.^2^^-^^4^ Review of literature revealed variably sized lesions of ectopic prostate tissue involving a variety of organs outside the urinary tract, including pericolic fat, anal canal, uterine cervix, spleen, and seminal vesicle.^2^^-^^9^ The ectopic location might be related to the abnormal embryogenesis or divergent differentiation of the prostate, rectum, and bladder.^3^

## CASE REPORT

**Fig.1:**
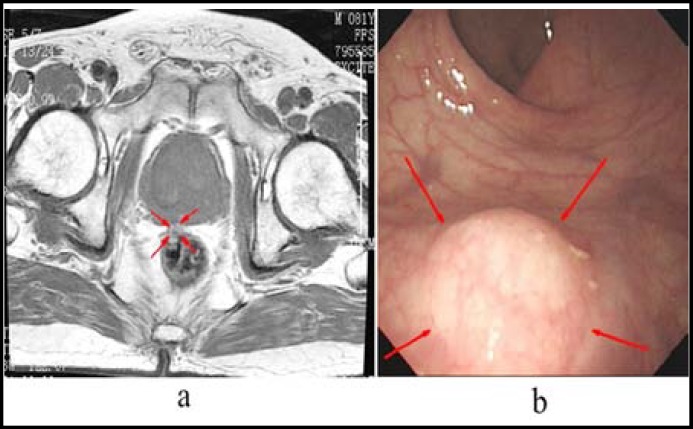
**a. **Colonoscopy shows the nodule (arrows), it was 1cm in diameter and in the anterior rectum 5cm above the anal verge with an otherwise normal look of the surface rectal mucosa. **b. **Axial T1-weighted MRI demonstrates a low intensity and well-circumscribed nodule (arrows) measuring 1cm in diameter at the level of 5cm above the anal verge protrude to the rectal lumen.

An 81-year-old man with a history of rectal bleeding for the previous one month was referred to our clinic. He presented bright blood which was not mixed with the stool. The patient who was otherwise healthy, had no previous history of urologic intervention. Digital rectal examination found a firm, round solid submucosa nodule in the anterior rectum, it was 1cm in size and 5cm above the anal verge. After administration, systemic examination showed normal results with normal level of serum cancer embryo antigen (CEA 2.38 ng/ml) and prostate specific antigen (free PSA 0.8 ng/ml, total PSA 2.38 ng/ml). Colonoscopy confirmed the nodule was 1cm in diameter and in the anterior rectum 5cm above the anal verge with an otherwise normal look of the surface rectal mucosa (Fig.1a). Magnetic resonance imaging (MRI) demonstrated a well-circumscribed and submucosa nodule measuring 1cm in diameter at the level of 5cm above the anal verge protrude to the rectal lumen (Fig.1b), with relatively low intensity on T1-weighted images and high intensity on T2-weighted images, however it was enhanced on the contrast imaging. The border between the nodule and the prostate was clear. After the diagnosis for a nodule in the submucosa rectum was made with the clinically suspicious of carcinoid tumor, local excision of the nodule en bloc with 5mm normal mucosa around the nodule and some superficial muscularispropriawas performed.

**Fig.2:**
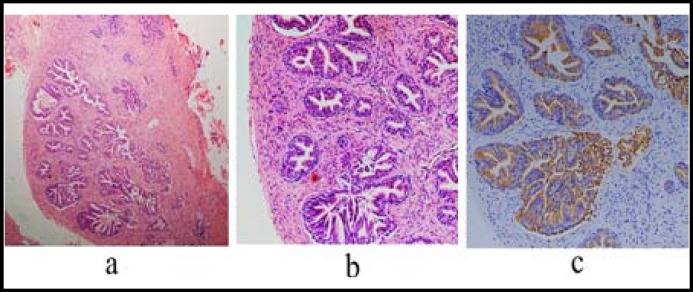
Submucosa ectopic prostatic tissue of the rectum: a and b, Low and high power view of hematoxylin eosin stain (a, 40×; b, 100×) a well-encapsulated submucosa nodule of dilated glandular structures surrounded by a dense fibrovascularstroma, with a basal layer and a luminal layer of cuboidal to columnar cells. Typical prostatic acini with papillary infoldings of the luminal epithelium and occasional cribriforming of the glands. c, Immunohistochemical staining for PSA (c, 100×) strongly highlights the glandular epithelium.

During the procedure a well-encapsulated round nodule was found in the submucosa. The nodule was 1cm in diameter, and could be separated from the surrounding submucosa tissue. Postoperative days were uneventful, and the patient was discharged on the second postoperative day. Under the microscope, the histologic sections revealed a well-encapsulated nodule of dilated glandular structures. The nodule was full of dilated glandular structures which was located between the severe burnt rectal mucosa and muscularispropria. The dilated glandular structures were surrounded by a dense fibrovascularstroma, which contained bland, spindled and ovoid cells without mitotic activity. Within the glandular epithelium, a range of morphologic appearances was evident and which showed the prostatic nature. The glands of interest had both a luminal layer and a basal layer of cuboidal to columnar cells. The typical prostatic acini possessed papillary infoldings of the luminal epithelium and occasional cribriforming of the glands, some degree of corpora amylacea was present (Fig.2a through 2c). Immunohistochemical staining for PSA strongly revealed positive staining of the epithelium (Fig.2c), confirming the prostatic nature of the nodule, while the stromal cells showed strong staining for actin and smooth muscle actin. No evidence of malignancy was observed. This patient has no evidence of recurrent disease during four years follow-up.

## DISCUSSION

The appearance of ectopic prostate tissue has been reported several times. Most cases occur in male urinary tract, as a recognized cause of asymptomatic hematuria, which are found most commonly in the prostatic urethra near the verumontanum.^5^ The presence of prostate tissue from the outside of urinary system is extremely rare. Ectopic prostate tissue has previously been reported in the lower gastrointestinal tract such as anal canal.^4^^,^^7^ However, this case is unique because it’s the first description of the presence of ectopic prostatic tissue in mural of the rectum. This is a highly unusual finding and probably reflects abnormal embryogenesis or divergent differentiation.^2^

Two hypothesizes for the potential mechanism of prostate tissue within rectal submucosa can be postulated from the normal embryologic origins of the prostate, bladder, and rectum, which may be explained on the basis of faulty embryogenesis. The rectum and bladder originate from the endodermal cloaca, which is a single hollow tube. This tube is divided into rectum and bladder at around 5th weeks of gestation by a caudal proliferation of mesoderm, the urorectal septum. The walls of the anterior portion fuse with the developing mesonephric ducts and form the bladder and urethra. Tubular outgrowths budding from the urethra eventually form the encircling prostate at the 12th week of gestation. While the dorsal compartment forms the rectum.^1^^,^^3^^-^^5^ Because of some failure of morphogenetic tissue organization in this case, when the endodermal cloaca was divided into rectum and bladder, the cells capable of differentiating into prostatic tissue had traveled with the rectum, so that some dorsal cloacal compartment cells retained the ability to produce anterior cloacal structures and later formed a distinct rectal submucusa ectopic prostatic tissue, which destined to differentiate to rectum.^1^^,^^3^ However, another hypothesis is that the cells capable of differentiating into prostatic tissue were part of the rectal wall which might have formed aberrant tubular outgrowths within the rectal submucosa to create the ectopic prostate.^1^

As most cases have been incidental findings and probably significantly underreported, the true incidence of the ectopic prostatic tissue outside the urinary tract has not been fully evaluated.^1^ Making a preoperative diagnosis for ectopic prostatic tissue outside the urinary tract is extremely difficult. Most cases were diagnosed postoperatively, even were incidental finding on surgical specimens without any relative clinical presentation.^5^^-^^7^ The imaging studies, such as CT, MRI, and ultrasound, as well as the endoscopy are easy to reveal big lesions, meanwhile CT or ultrasound-guided biopsy can be performed^8^,but non-effective for small ones. Histological sections of the specimens revealed the typical histological features of prostatic acini and stroma. 

The dual cell layers (columnar epithelial cells and basal cells) are the key to recognize the ectopic prostatic tissue. Besides, the presence of corpora amylacea is distinctive, which can be very helpful for diagnosis.^1^ When this kind of tissue is encountered, PSA is usually the first immunohistochemical stain to be used, which is highly specific and sensitive marker for tissue of prostatic origin. For the cases that PSA staining are negative, other makers for the prostatic tissue are recommended to be used in conjunction with it. Prostate-specific acid phosphatase (PSAP) and P501S are preferred, and so far the latter one has been detected only in prostatic tissue in males.^1^ If the above stains are inconclusive, the basal cell markers such as p63 and high molecular weight cytokeratins (HMWCK, 34βE12), or a PIN4 cocktail (P504S, p63, and HMWCK), are recommended to demonstrate the presence of basal cells and secretory epithelial cells.^1^^,^^2^

The presence of ectopic prostatic tissue outside the urinary tract is important in several respects, and it can raise clinically important issues, depending on the location. It can be a cause of rectal bleeding or bowel obstruction symptoms. In addition, it may be confused with malignancy in the gastrointestinal system, or retrovesical space.^3^^,^^4^ Usually, surgical treatment by simple extirpation of the tumor is recommended and is curative.^9^ There is possibility that ectopic prostatic tissue malignant transformation can happen^10^, however microscopic examination of the specimen in our case has not revealed any sign of malignancy.

In summary, we have reported the first case of ectopic prostatic tissue within the submucosa of rectum. This is a highly unusual finding and probably reflects abnormal embryogenesis. Ectopic prostatic tissue in this location may be an underrecognized condition, however it has a characteristic histologic appearance and the diagnosis is easily confirmed using immunohistochemistry. This patient was cured by simple excision, and had no recurrence during the four years after the surgery.
